# Microscopic examination and smear negative pulmonary tuberculosis in Ethiopia

**DOI:** 10.11604/pamj.2014.19.162.3658

**Published:** 2014-10-16

**Authors:** Tibebe Seyoum Keflie, Gobena Ameni

**Affiliations:** 1 Ethiopian Society of Tropical and Infectious Diseases, Ethiopia; 2 Aklilu Lemma Institute of Pathobiology, Addis Ababa University, Ethiopia; 3 College of Medicine and Health Sciences of Mada Walabu University, Ethiopia

**Keywords:** Microscopic examination, smear negative pulmonary TB, sensitivity, specificity

## Abstract

**Introduction:**

Tuberculosis causes illness among millions of people each year and ranks as the second leading cause of death from infectious disease worldwide. The aim of this study was to investigate the detection rate of microscopic examination and estimate risk of transmission of TB by smear negative pulmonary TB patients.

**Methods:**

A cross-sectional study and retrospective data analysis on TB were undertaken in Northwest Shewa, Ethiopia. Microscopic examination, bacterial culture and PCR were performed. The statistical analysis was made by using STATA software version 10.

**Results:**

A total of 92 suspected TB cases was included in the study. Of these, 27.17% (25/92) were positive for microscopic examination and 51% (47/92) for culture. The sensitivity and specificity of microscopic examination with 95% CI were 48.94% (34.08% to 63.93%) and 95.56% (84.82 to 99.33%), respectively. The positive and negative predictive values were 92% (73.93% to 98.78%) and 64.18% (51.53% to 75.53%), respectively. Of 8150 pulmonary TB cases in the retrospective study, 58.9% was smear negative. The proportion of TB-HIV co-infection was 28.66% (96/335).

**Conclusion:**

The sensitivity of microscopic examination was 48.94% which was very low. The poor sensitivity of this test together with the advent of HIV/AIDS elevated the prevalence of smear negative pulmonary TB. This in turn increased the risk of TB transmission.

## Introduction

Tuberculosis (TB) still remains a major global public health problem with 8.6 million incidence and 1.3 million deaths in 2012 [1]. TB causes illness among millions of people each year and ranks as the second leading cause of death from infectious disease worldwide, after the human immunodeficiency virus (HIV) [1]. The majority of people affected by TB are found in economically poor countries where sputum microscopy with a conventional light microscope is the primary method for diagnosing pulmonary TB [2]. In the early 1990s, Multi drug resistant (MDR) - TB strains emerged and have now been found all over the world [3]. Most recently, the global concerns about the emergence of MDR-TB and extensively drug resistant (XDR)-TB have emphasized the need to tackle TB more effectively all over the world [4].

In 2010 / 11, Ethiopia undertook the national population based TB survey which is the first in Africa. The survey report revealed that the prevalence of smear positive TB among adults and all age groups was found to be 108 and 63 per 100, 000 populations, respectively [5]. The prevalence of bacteriologically confirmed TB was found to be 156 per 100,000 populations [5]. Moreover, Ethiopia is one of the 27 high MDR-TB burden countries [1]. Microscopic examination is rapid, relatively simple, inexpensive and highly specific [2]. Laboratory diagnosis of TB is usually done in Ethiopia using acid fast bacilli (AFB) smear microscopic examination [5].

The threshold for detection of AFB in sputum samples under optimal conditions is between 10^4^ and 10^5^ bacilli per ml [6]. However, the infecting dose of *Mycobacterium tuberculosis* bacilli is estimated to be fewer than ten organisms [7]. This imply that microscopic examination of AFB identifies the most infectious and misses the less infectious patients. Microscopically missed TB cases are considered to be smear negative. However, patients with smear negative culture positive TB appear to be responsible for about 17% of TB transmission [7]. Therefore, the aim of this study was to investigate the detection rate of microscopic examination of sputum smear and estimate the risk of transmission of TB by smear negative pulmonary TB patients in Ethiopia.

## Methods

The study was conducted in Fiche Hospital, North Shewa Zone of Oromia Regional State, Ethiopia. A cross-sectional study design and retrospective data analysis were used. The data were collected in 2007. A total of 92 TB suspected patients were included for the investigation of the detection rate of microscopic examination. In this case, the Suspected TB cases are those with symptoms and signs of suggestive of TB, in particular cough of two weeks or more duration [8]. Furthermore, five years TB case report data of the zone were used to estimate the risk of transmission of TB from microscopically missed smear negative pulmonary TB patients.

### Diagnosis of TB

**Figure 1:**
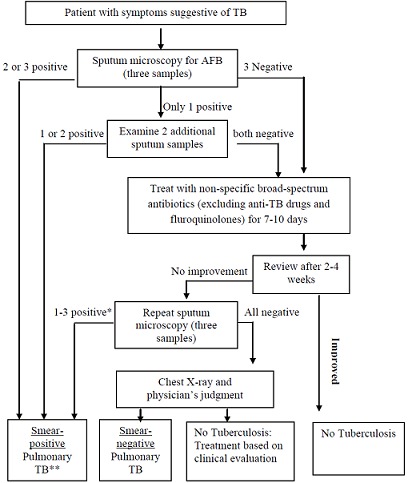
Diagnostic algorithm of pulmonary TB adopted from FMOH, 2013

National standard diagnostic algorithm indicated by Figure 1 is used for the diagnosis of TB cases in Ethiopia. Microscopic examination is used for diagnosis, monitoring and defining cure rate of treatment. Three sputum specimens must be collected and examined in two consecutive days (**spot-early morning-spot**) [5].

### Definition of different TB cases

Based on the national guideline, the following TB case classifications were defined [5].

*Smear-positive pulmonary TB (PTB + ):* a patient with at least two initial sputum smear examinations positive for AFB by direct microscopy, or a patient with one initial smear examination positive for AFB by direct microscopy and culture positive, or a patient with one initial smear examination positive for AFB by direct microscope and radiographic abnormalities consistent with active TB as determined by a clinician.

*Smear-negative pulmonary TB (PTB-):* a patient having symptoms suggestive of TB with at least 3 initial smear examinations negative for AFB by direct microscopy, **and** 1. No response to a course of broad-spectrum antibiotics, 2. Three negative smear examinations by direct microscopy, 3. Radiological abnormalities consistent with pulmonary TB, 4. Decision by a clinician to treat with a full course of anti- TB **or** a patient whose diagnosis is based on culture positive for M. tuberculosis but three initial smear examinations negative by direct microscopy.

### Sputum collection and transportation

All suspected TB patients in the out patient department (OPD) of Fiche Hospital were invited to participate in the study. Orietation was given to participating individuals regarding the difference between sputum and saliva. They were also told to collect their sputum for two consecutive days, Spot - Early Morning - Spot. The patients were also told to collect their sputum in well aerated places to a volume of 5-10ml during productive cough in separate plastic caps; to tightly close the lid of the cap and disinfect it with tuberculocidal disinfectants. All specimens were kept at 4°C and transported to Aklilu Lemma Institute of Pathobiology, Addis Ababa University for mycobacterial culture using cool ice box.

### Microscopic examination of AFB smears

AFB smears were prepared using **Ziehl-Neelsen** staining technique in Fiche Hospital laboratory. The smears were air dried; flooded with carbol fuchsin; heated gently until steam came out; washed with fine jet of water; decolorized with acid alcohol; counter stained with methylene blue for 1 minute after washing acid alcohol in the same way; air dried (blot dried) and finally examined for minimum of 100 oil immersion fields.

### Mycobacterial culture

The sputum specimens were decontaminated by 4% sterile NaOH solutions, mixed very well and centrifuged at 3000rpm for 10 minutes. The supernatant of each specimen was decanted and the sediment was resuspended with normal saline. A drop of phenol red indicator was added to the suspension and 2N HCl was added drop by drop until the colour changed to yellow. Two Lowenstein-Jensen (LJ) slants, one with pyruvate and the other with glycerol were prepared for each specimen and inoculated with 1 ml or three to four drops of inoculums. Inoculated tubes were incubated in a slanted position with the tube′s screw cap loosened for at least the first week at 37°C in an atmosphere of 10% CO_2_ and 90% air, and then vertical position upto 8 weeks for the better development of individual colonies.

### Polymerase Chain Reaction (PCR)

For extraction and amplification of DNA from mycobacterial cells, a fresh culture was suspended in 100 to 200µl of distilled water and incubating them at 85°C for 30 min, and then centrifuged for 30 seconds at 13,000g. A volume of 50µl of the supernatant was then used for the PCR procedures. The primers used in PCR were RD10 flankF CTGCAACCATCCGGTACAC; RD10intR GAAGTCGTAACTCACCGGGA; and RD10flankR AAGCGCTACATCGCCAAG. If RD 10 is present (i.e. *M. Tuberculosis*), a product size of 380bp (RD 10int + RD 10 flankR) will be obtained; if it is deleted (*M.africanum and M. Bovis*); then product of 202 bp (RD10 flankF + RD10 flankR) will be obtained. A hot start master mix (STRTAGENE ^®^, USA) was used. PCR amplifications with mixtures containing, per reaction, 1.25µl of 10x PCR buffer (600mM Tris HCl (pH 8.8), 20mM nucleotide mix, 50nM each primer, 1 to 10ng of template DNA, 10% dimethyl sulfoxide, 0.2U of Taq polymerase (Gibco BRL), and sterile destilled water to 12.5 µl were performed on a PTC-100 amplifier (MJ Inc.) with initial denaturation step of 90 seconds at 95oC followed by 35 cycles of denaturation for 30s at 95°C, annealing for 1 min at 58°C, and extension for 4 min at 72°C. Final extension was performed at 72°C for 10 min. After amplification, a 10 µl of the PCR product was analyzed by 7.5% polyacrylamidogel electrophoresis. Electrophoresis was carried at a constant voltage of 200 for 45 min using a vertical min protean II™apparatus (Biorad USA) filled with 1 X TBE buffer. The gel was then stained by the silver nitrate method described by Cleaveland et al. (2007) [9] and the gel preserved at 4°C in a sealed nylon bags to avoid drying.

### Retrospective analysis of TB reports

Data of TB were compiled from the reports given to TB and Leprosy control program of the infectious diseases control unit of North Shewa Health Bureau. The reports were between 2002 and 2007. TB - HIV co-infection report was started at Fiche Hospital in 2007. The data on TB-HIV co-infection were those which were summarized from Fiche Hospital TB Clinic record book.

### Ethical consideration

The study was ethically approved by the institutional ethical review board of Aklilu Lemma Institute of Pathobiology, Addis Ababa University. Besides, informed written consent and assent were obtained from each of study participants prior to enrolment.

### Data entry and analysis

Statistical analysis was done by STATA software version 10. Sensitivity, specificity, positive predictive value and negative predictive value of microscopic examination was calculated and described as percentage with 95% confidence interval. The agreement between microscopic examination and mycobacterial culture was indicated by using Kappa statistics. Descriptive statistics were used to summarize the retrospective data for the estimation of the risk of TB transmission from smear negative TB cases.

## Results

### Microscopic examination

Based on suggestive clinical signs and symptoms, 92 new TB cases were included in the study between December and January, 2007. Of the 92 cases, 27.17% (25/92) were positive for microscopic examination of sputum smear and 51% (47/92) was mycobacterial culture positive (Table 1). Taking mycobacterial culture as gold standard, sensitivity, specificity, positive predictive value (PPV) and negative predictive value (NPV) of microscopic examination was calculated at 95% confidence interval. The sensitivity and specificity were 48.94% (34.08% to 63.93%) and 95.56% (84.82% to 99.33%), respectively. The positive and negative predictive values of this test were 92% (73.93% to 98.78%) and 64.18% (51.53% to 75.53%), respectively. A substantial agreement (k = 0.7023) was recorded between microscopic examination and mycobacterial culture.

**Table 1:** Microscopic sputum smear examination and mycobacteriological cultur results

Sputum smear	Mycobacteriological cultur		
	Positive	Negative	Total
Positive	23	2	25
Negative	24	43	67
Total	47	45	92

### Polymerase Chain Reaction (PCR) results

The type of *M. tuberculosis* complex was identified by mycobacterial culture and confirmed by PCR. In this study, fifteen culture positive specimens were analysed by PCR and 14 of them were confirmed as *M. tuberculosis*.

### Retrospective data analysis

### Trend of TB distribution

**Figure 2:**
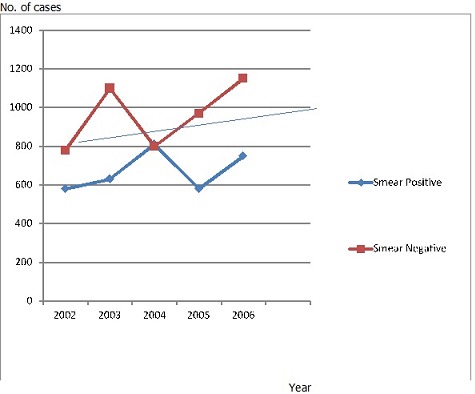
Trend of smear positive and negative pulmonary tuberculosis cases in Northwestern Shewa between 2002 and 2006

**Figure 3:**
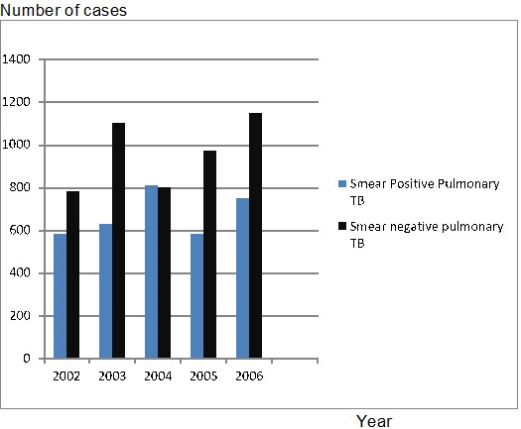
Burden of smear positive and negative pulmonary TB in Northwestern Shewa

The distribution of smear positive and smear negative pulmonary TB cases in North Shewa zone of Oromia Regional State, Ethiopia between 2002 and 2007 was analysed. During these periods, 8150 pulmonary TB cases were reported. Of these, 41.10% (3350/8150) was smear positive and 58.9% (4800/8150) was smear negative pulmonary TB. The numbers of smear positive and negative TB cases showed slight fluctuation during these periods of time but their overall trends as indicated in Figure 2 were rising up. The incidence of smear negative TB case was the highest in all years except 2004 (Figure 3). Population age groups between 14 to 34 years were highly affected in the study area between 2002 and 2007. More than 87.3% of TB patients were between 14 to 54 years of age. Similarly, 60.9% (4963/8150) males and 39.1% (3187/8150) females were affected by pulmonary TB during these periods of time in the study area.

### TB-HIV co-infection

TB-HIV co-infection report started in the study area in 2006. In this year, 335 TB patients were tested for HIV and 96 of them were found positive in Fiche Hospital. The proportion of co-infection was 28.66% (96/335).

## Discussion

Sputum microscopy is the mainstay of diagnostic methods in Ethiopia. It is simple, cheap and very fast technique which is highly specific in areas with a very high prevalence of TB [10]. Although it has high specificity, its detection rate is very low. Of 92 TB suspected cases in this study, 27.17% (25/92) was positive by microscopic examination whereas 51% (47/92) was positive by mycobacterial culture. The sensitivity and specificity of microscopic examination with 95% confidence interval were 48.94% (34.08% to 63.93%) and 95.56% (84.82% to 99.33%), respectively. The sensitivity of this study was closely similar to the report of Greenbaum, et al., 1980 (48.10%) [11]. However, it was by far smaller than the reports of Warren et al., 2000 (92%) [12], Zar, 2004 (60%) [13], Marie, et al., 1995 (81.6%) [14] and Uddin, et al., 2013 (70.5%) [6]. On the other hand, the sensitivity of this study was higher than the report of Peterson, et al., (28%) [15] and, Toman and Frieden, 2005 (22 - 43%) [16].

Despite of the wide application as primary diagnostic tool, the detection power of microscopic examination is not consistent. This is justified by paucibacillary nature of the disease, quality and quantity of sputum, skill of microscopist, smear preparation, lack of patience and exhaustion of lab technicians due to high load. The other disadvantages of microscopic examination is its inability to diagnose EPTB, paediatric TB and in individual who is co-infected with HIV-TB [17].

According to National standard diagnostic algorithm (Figure 1), suspected TB cases who are not positive by microscopic examination are followed up for at least 2 to 4 weeks with non-specific broad-spectrum antibiotics treatment (excluding anti-TB drugs and fluroquinolones) [5]. After a while, the patients are diagnosed either as smear positive, smear negative or non TB case with improved health. This implied that each of the suspected TB cases should at least wait for fourteen and more days before commencing anti-TB drug regimen as the result of low sensitivity of microscopic examination. In fact, less than ten M. tuberculosis bacilli are required to get TB infection [18] and these patients can expel even more organisms at a time. This in turn implied that in the due process of diagnosis, the suspected cases propagate TB infection to considerable number of populations in the community.

Marcel, et al., (1999) reported that patients with smear-negative culture-positive TB appear responsible for about 17% of TB transmission [7]. The epidemiological contribution of smear-negative patients to TB transmission has implications for TB - control strategies in developing countries where such patients are usually overlooked [7]. About 46.81% of culture positive TB patients in this study was smear negative which was a bit higher than the reports of Belete, et al., 2013 (33.9%) [19] and Desta, et al.,2009 (17.4%) [20].

Between 2002 and 2006, 8150 pulmonary TB cases were reported in Northwest Shewa, Ethiopia. Of these, 58.9% (4800/8150) was smear negative pulmonary TB. During these periods of time, the prevalence of smear negative TB was increasing (Figure 2). In HIV infected TB patients, the proportion of smear-negative pulmonary TB patients was high [21]. In this study, the proportion of TB-HIV co-infection was 28.66% (96/ 335). This result was smaller than the findings of Gellete et al., 1997 (44.4%) [22] in Shashamene General Hospital, Southern Ethiopia and Demisse et al., 2001 (45.3%) [23] in Addis Ababa. However, it was larger than 20.5% and 22% incidences which were reported by Madebo et al., (1997) [24] in Yirga Alem Hospital in Southern Ethiopia and Mitike et al., (1997) [25] in Harrar Hospital in Eastern Ethiopia, respectively. Low sensitivity of microscopic examination together with the advent of HIV/AIDS increased the prevalence of smear negative pulmonary TB. From the experience, smear negative pulmonary TB patients take less precaution to prevent TB transmission. And hence, increasing the prevalence of smear negative pulmonary TB increased the risk of TB transmission

Similarly, the age and sex distribution of pulmonary TB in this study showed that more than 87.3% population between 14 and 54 years of age, and 60.9% males were affected between 2002 and 2006 in the study area. This indicated that pulmonary TB affected more productive forces so that it has both public health and economic implication. Therefore, improving the detection rate of microscopic examination and reducing the burden of smear negative pulmonary TB are the priority issue in the TB control strategies.

## Conclusion

The sensitivity of microscopic examination was 48.94% which was very low. The poor sensitivity of this test together with the advent of HIV/AIDS elevated the prevalence of smear negative pulmonary TB. This in turn increased the risk of TB transmission. Therefore, improving the detection rate of microscopic examination or designing very rapid, highly sensitive, cheap and friendly useable diagnostic tool is the priority research area in TB control program.
